# Statistical Analysis of the Role of Cavity Flexibility in Thermostability of Proteins

**DOI:** 10.3390/polym16020291

**Published:** 2024-01-21

**Authors:** So Yeon Hong, Jihyun Yoon, Young Joo An, Siseon Lee, Haeng-Geun Cha, Ashutosh Pandey, Young Je Yoo, Jeong Chan Joo

**Affiliations:** 1 Department of Chemical and Biological Engineering, Inha Technical College, Inha-ro 100, Michuhol-gu, Incheon 22212, Republic of Korea; soyeon.hong@inhatc.ac.kr; 2 Department of Biotechnology, The Catholic University of Korea, Bucheon-si 14662, Republic of Koreasiseon@catholic.ac.kr (S.L.);; 3 Institute for Water and Wastewater Technology, Durban University of Technology, 19 Steve Biko Road, Durban 4000, South Africa; ashutoshkscem@gmail.com; 4 Department of Biotechnology, Faculty of Life Science and Technology, AKS University, Satna 485001, Madhya Pradesh, India; 5 School of Chemical and Biological Engineering, Seoul National University, Seoul 08826, Republic of Korea; yjyoo@snu.ac.kr

**Keywords:** protein thermostability, cavity, flexibility, thermophilic proteins, mesophilic proteins, statistical analysis

## Abstract

Conventional statistical investigations have primarily focused on the comparison of the simple one-dimensional characteristics of protein cavities, such as number, surface area, and volume. These studies have failed to discern the crucial distinctions in cavity properties between thermophilic and mesophilic proteins that contribute to protein thermostability. In this study, the significance of cavity properties, i.e., flexibility and location, in protein thermostability was investigated by comparing structural differences between homologous thermophilic and mesophilic proteins. Three dimensions of protein structure were categorized into three regions (core, boundary, and surface) and a comparative analysis of cavity properties using this structural index was conducted. The statistical analysis revealed that cavity flexibility is closely related to protein thermostability. The core cavities of thermophilic proteins were less flexible than those of mesophilic proteins (averaged B’ factor values, −0.6484 and −0.5111), which might be less deleterious to protein thermostability. Thermophilic proteins exhibited fewer cavities in the boundary and surface regions. Notably, cavities in mesophilic proteins, across all regions, exhibited greater flexibility than those in thermophilic proteins (>95% probability). The increased flexibility of cavities in the boundary and surface regions of mesophilic proteins, as opposed to thermophilic proteins, may compromise stability. Recent protein engineering investigations involving mesophilic xylanase and protease showed results consistent with the findings of this study, suggesting that the manipulation of flexible cavities in the surface region can enhance thermostability. Consequently, our findings suggest that a rational or computational approach to the design of flexible cavities in surface or boundary regions could serve as an effective strategy to enhance the thermostability of mesophilic proteins.

## 1. Introduction

Protein thermostability is one of the major factors affecting industrial applicability and engineering thermostability of proteins that has been rigorously studied [1,2,3]. The statistical studies of thermophilic and mesophilic proteins have attempted to understand major structural features that govern protein thermostability. According to previous results [4,5], thermophilic proteins favor electrostatic interactions at the surface or tight packing in the core. Moreover, thermophilic proteins are less flexible than mesophilic proteins at high temperatures; normally, thermophilic proteins are stable but are not active at low temperatures [6]. Rational design studies, based on rules revealed by statistical approaches, have been performed to improve the protein thermostability but their stabilization effects are case-by-case, indicating that there are no general rules to engineer protein thermostability [7]. However, among these rules, increasing core packing has been considered an effective and generally applicable rationale to improve protein thermostability [8,9,10]. Moreover, a recent packing study [11] revealed that external residues of thermophilic proteins had better packing than mesophilic proteins. In the case of packing enhancement, Gly to Ala or Ala to Val mutations were conducted but the selection of target residues was dependent on the researcher’s insight; thus, thermostabilization effects were not always positive [12].

In the consideration of vacant space in protein structure, packing and cavity were often indiscriminately used for protein thermostabilization. However, a cavity is an interior empty space that is not accessible to the solvent probe and is normally detected by a water probe with a radius of 1.4 Å. The protein cavity is closely related to the enzyme dynamics and important for enzyme functions as well as stability [9,13]. Compared to qualitative selection criteria for core packing, the protein cavity can be quantitatively identified and can be aimed to target protein thermostabilization due to clear structure definition. A cavity-filling method is the most popular approach to engineering protein cavities for protein thermostabilization.

**Figure 1:**
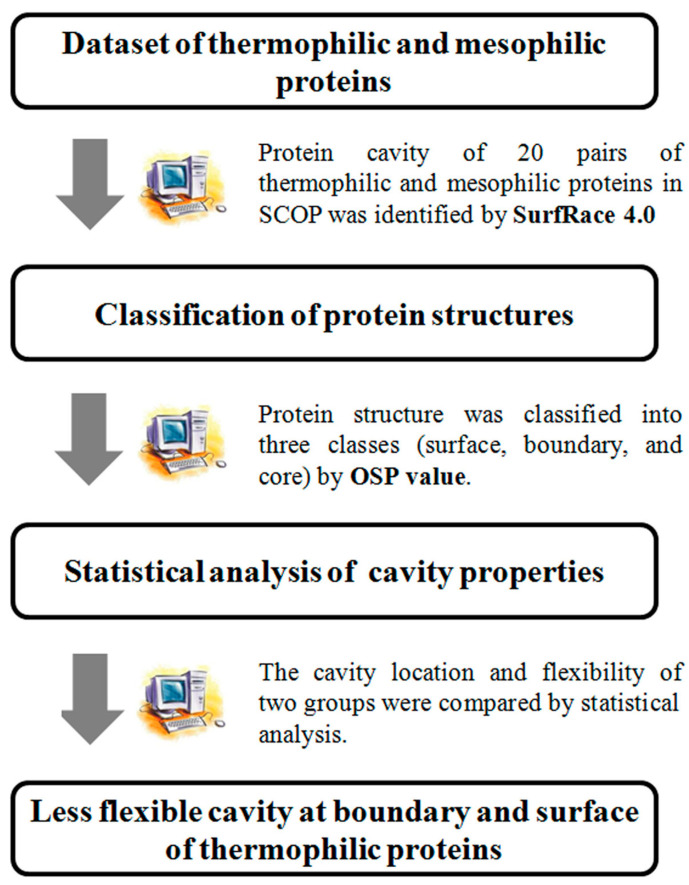
Scheme of statistical analysis of cavity properties in this study.

Statistical studies of differences in cavity properties between thermophilic and mesophilic proteins were also performed to investigate their role in protein thermostability, but no striking differences in cavity properties were found [14,15]. However, these results were based on a simple comparison of the volume, area, and number of cavities and neglected the important cavity properties related to the protein thermostability such as flexibility or location in three-dimensional structures. In this study, the cavity location and flexibility of homologous thermophilic and mesophilic proteins were compared by *t*-tests to investigate the role of these cavity properties in the protein thermostability (Figure 1). Three-dimensional structures were classified into three areas, i.e., surface, boundary, and core, using the OSP (occluded surface packing) value [16]. The flexibility of the cavity was calculated by normalized B factor values. Then, the location and flexibility of cavities in thermophilic and mesophilic proteins were compared. Examples of engineering the flexible cavities in the surface areas for the enhancement of protein thermostability were also discussed. This study elucidates the significance of cavity properties, namely their location and flexibility, in determining protein thermostability.

## 2. Materials and Methods

### 2.1. Dataset of Homologous Thermophilic and Mesophilic Proteins

The dataset of homologous thermophilic and mesophilic proteins was adapted from Yokota’s work [17]. Yokota’s dataset has no fold redundancy and can be useful to investigate cavity properties in various folds. The number of protein cavities is proportional to the size of the protein; thus, small proteins have only a few cavities. To obtain reliable results, protein pairs with a small size (<200 amino acids) or low homology (<35%) were removed from Yokota’s dataset. A total of 20 protein pairs were used for statistical analysis (Table 1). The crystal structures of homologous thermophilic and mesophilic proteins from the Protein Data Bank were further optimized by energy minimization using conjugate gradient algorithms within Discovery Studio 2.5 (Accelrys, San Diego, CA, USA), as performed in previous studies [18].

### 2.2. Structure Classification by Residual Packing Value

**Figure 2:**
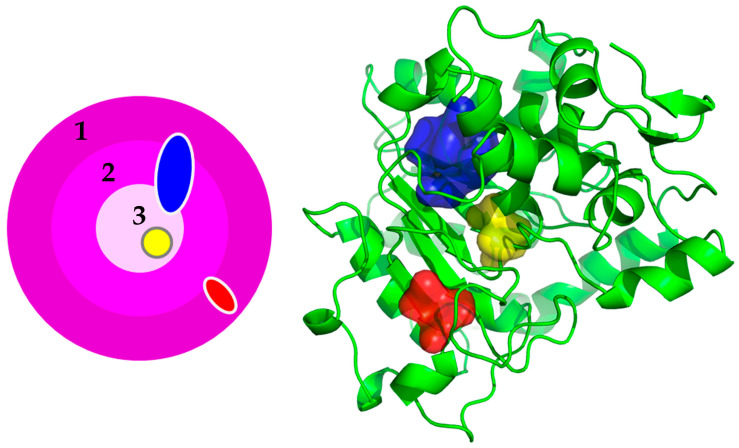
Simplified scheme for cavity location in the three-dimensional structure. Cavities in surface (1), boundary (2), and core (3) are shown in red, blue, and yellow, respectively. The blue cavity is positioned along all three areas.

The structure index proposed by Pack [5] was used to calculate the exact cavity location in three-dimensional structures. The OSP value of proteins was calculated by the occluded surface algorithm [19]. Because the protein cavity consists of at least three amino acids and cavity-lining residues in one cavity can have a wide distribution in protein structure (Figure 2), the three-dimensional structure after energy minimization was broadly divided into three classes by the OSP value, i.e., surface (0.000~0.250), boundary (0.250~0.500), and core (0.500~0.750), instead of the original five classes. The OSP value of each cavity was determined by averaging the OSP values of cavity-lining residues.

### 2.3. Calculation of Cavity Flexibility

The protein cavity was identified by SurfRace 4.0 [20] software with a 1.4 Å probe. B factor value was used as a flexibility indicator of the protein cavity. The experimental B factor value is quite dependent on the structure resolution or crystal contacts; thus, it should be normalized to compare different structures [21]. B factor values of Cα atoms for cavity-lining residues were normalized using Equation (1):

B′ = (B − <B>)/σ(1)

where B is the actual B factor value, <B> is the average B factor value in a given chain, σ is the standard deviation of B factor values for all Cα atoms in a given chain, and B′ is the normalized B factor value.

### 2.4. Statistical Analysis

A *t*-test was conducted to estimate quantitative differences in cavity location and flexibility between thermophilic and mesophilic proteins. All statistical analyses were performed as described in previous studies [5,17]. The *t*-test parameter (t_i_) can be calculated using Equation (2):

t_i_ = (X_i-Th_ − X_i-Me_)/√(S^2^_i-Th_/N_Th_ + S^2^_i-Me_/N_Me_) (2)

where S^2^_i-Th_ and S^2^_i-Me_ are the deviations of average traits, X_i_ in structure index I, of thermophilic and mesophilic proteins, respectively; and N_Th_ and N_Me_ are the total number (20 proteins in each group) of thermophilic and mesophilic proteins, respectively.

Here, the degrees of freedom, df (= N_Th_ + N_Me_ − 2), are 38, which values are sufficient to be considered as infinite sample sets. For a one-tailed *t*-test (with df > 30), the critical levels of the *t* value are as follows (Table 2) [22].

If t_i_ > 1.282, then the probability that average frequencies, X_i_, of thermophilic protein groups are greater than X_i_ of mesophilic protein groups if the structure state i is >0.90. In contrast, if t_i_ < −1.282, then the probability that average frequencies, X_i_ of thermophilic protein groups is less than X_i_ of mesophilic protein groups if the structure state i is >0.90 [5].

## 3. Results and Discussion

### 3.1. Comparison of Cavity Properties in Thermophilic and Mesophilic Proteins

**Figure 3:**
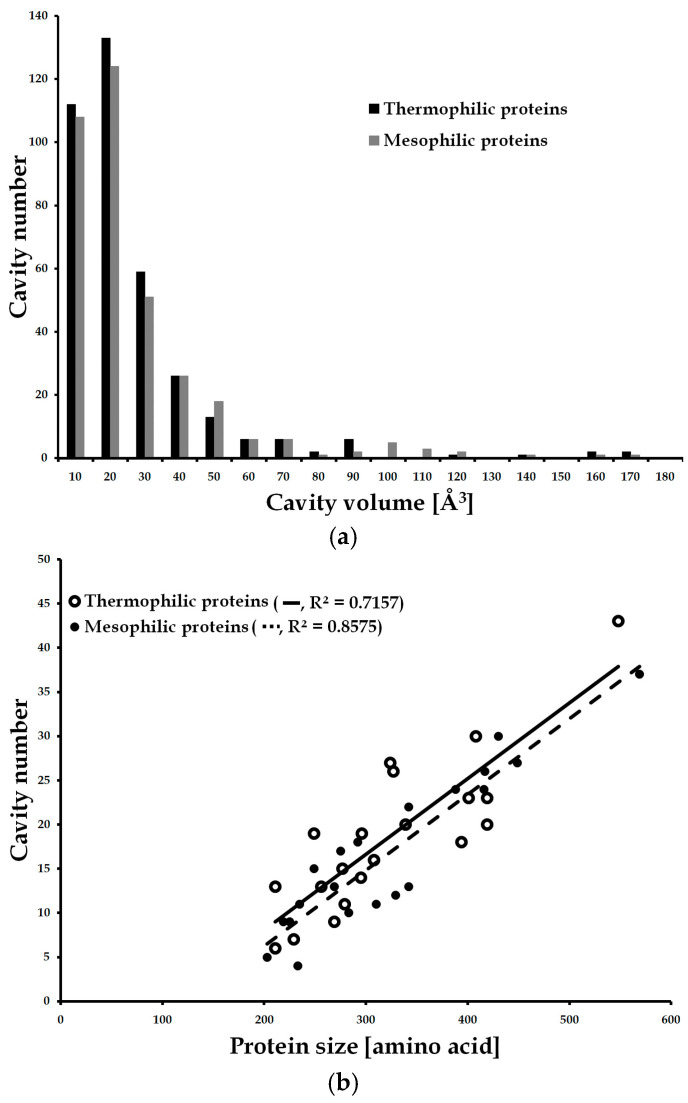
Comparison of cavity number and volume in thermophilic and mesophilic proteins. (**a**) Distribution of cavity number according to the protein size. (**b**) Distribution of cavity volume.

The cavity properties of thermophilic and mesophilic proteins, i.e., number, volume, and surface area were compared. The protein size of the dataset varied from 200 to 600 amino acids and both proteins showed a similar tendency in cavity number according to the protein size (Figure 3). Large proteins in both groups had more cavities, but thermophilic and mesophilic proteins showed differences in cavity volume. Thermophilic proteins had more small cavities (<~30 Å^3^), but mesophilic proteins favored large cavities (>~50 Å^3^) (Figure 3b). This might indicate that thermophilic proteins use smaller cavities for dynamic movements such as ligand binding or enzyme catalysis; conversely, mesophilic proteins have bigger cavities, which is advantageous to protein functions but deleterious to protein stability [9].

Contrary to the previous study [15], thermophilic proteins had more cavities than mesophilic proteins (369 and 355) and their cavity number per protein was slightly higher than that of mesophilic proteins (18.45 and 17.75) (Table 3). However, the protein size of thermophilic proteins was slightly bigger than mesophilic proteins (6638 and 6346); thus, the cavity number per residue of both groups was identical (0.056 and 0.056). Moreover, the volume and surface area of the cavity of mesophilic proteins were larger than those of thermophilic proteins, implying that the cavity volume, rather than the number, may be related to the protein thermostability. Thermophilic and mesophilic proteins showed differences in cavity volume, but this result was based on a simple comparison of averaged data. Statistical analysis, such as a *t*-test, is necessary to obtain more reliable results.

### 3.2. Difference in Cavity Location and Flexibility between Thermophilic and Mesophilic Proteins

To further understand the role of the cavity in the protein thermostability, the differences in cavity location and flexibility between thermophilic and mesophilic proteins were investigated using a *t*-test (Table 4). Contrary to amino acids, a single cavity can be often widely distributed inside of the protein (blue cavity in Figure 2). Narrow structure classifications, such as the five indexes proposed by Pack [5], cannot reflect properly cavity location in the three-dimensional structure. Protein structure was categorized into three indices, i.e., index 1 (surface), index 2 (boundary), and index 3 (core). The index 1, 2, and 3 had 0.000~0.250, 0.250~0.500, and 0.500~0.750 of the OSP value, respectively. The core and surface indicate fully buried and exposed states, respectively. The boundary connects the core and surface and thus partially buried and exposed states.

**Figure 4:**
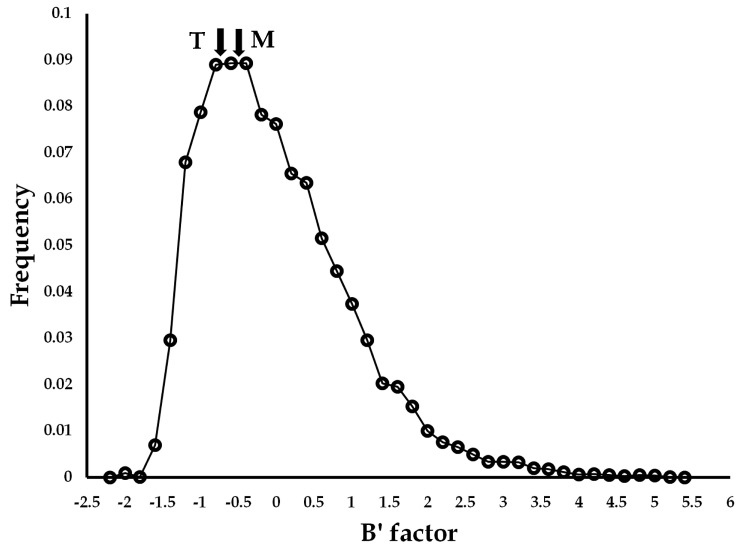
Averaged B′ factor of core cavities of thermophilic and mesophilic proteins. The open circle indicates the normalized B factor of all amino acids of 40 proteins. T and M indicate averaged B′ factor of core cavities of thermophilic and mesophilic proteins (−0.6484 and −0.5111), respectively.

According to the OSP value analysis, mesophilic proteins had more cavities in surface and boundary areas than thermophilic proteins. Based on the *t*-test analysis, there are statistically significant differences (>95% probability) in cavity location showing that thermophilic proteins preferred the core cavity, but mesophilic proteins had more cavities in boundary areas. In the case of the flexibility analysis, cavity-lining residues of thermophilic proteins were less flexible than those of mesophilic proteins in all locations, consistent with the known notion that mesophilic proteins are more flexible and less stable than thermophilic proteins at high temperatures [6]. Although the core cavity of both groups was relatively rigid in the distribution of the normalized B factor values of 40 proteins (12,974 amino acids), the core cavities of thermophilic proteins were less flexible than those of mesophilic proteins (−0.6484 and −0.5111), which might be less deleterious to protein thermostability (Figure 4).

According to the quantitative analysis of the location of cavity-lining residues and their flexibility, it can be concluded that mesophilic proteins preferred flexible cavities in surface and boundary areas, but thermophilic proteins favored rigid cavities in the core area. This result is similar to Glyakina’s work [11] showing that the packing of external residues is important to the protein thermostability. However, it is interesting that here, thermophilic proteins had more cavities in core regions, which is contrary to the previous results that thermophilic proteins had more packing and fewer cavities in the protein core [5,15]. In general, crystal structures of mesophilic and thermophilic proteins are determined at ambient temperatures, and they may not provide detailed insights into the molecular dynamics of proteins at extreme temperatures. Recent molecular dynamics simulations of mesophilic and thermophilic proteins revealed that thermophilic proteins exhibit greater flexibility than their mesophilic counterparts at elevated temperatures [23]. The conformational flexibility of thermophilic protein may facilitate the binding of a higher number of conformational substates [24]. While additional molecular dynamics simulations of mesophilic and thermophilic proteins are necessary, it is plausible that rigid cavities in the core regions of thermophilic proteins are linked to molecular motions at high temperatures.

Compared to mesophilic proteins, less flexible and fewer cavities of thermophilic proteins in the boundary and surface can be beneficial to the protein thermostability. However, more cavities of thermophilic proteins in the core could be deleterious to the protein thermostability. Conceptually, the protein cavity is bigger than residual packing in volume scale and the role of the protein cavity in protein structure should be understood in both aspects of function and stability. Thermophilic proteins are not active due to their structural rigidity at low temperatures but become active at high temperatures due to improved flexibility in elevated temperatures. In this study, thermophilic proteins had more cavities in the core, but these cavities were not flexible, indicating that thermophilic proteins might use core cavities for dynamic movements at high temperatures. According to previous comparative studies [4,25], thermophilic proteins have a high number of salt bridges at their surface to retain their structural stability. In particular, the force of salt bridges becomes stronger at high temperatures due to the decreased dielectric constant. The strength of electrostatic interactions is dependent on the distance and the dielectric constant. The shorter distance can contribute to stronger electrostatic interactions. Electrostatic interactions of less than 4 Å are typically called ion pairs or salt bridges. Media have different dielectric constants, e.g., 2 for n-hexane, 3.4 for n-octanol, 25 for ethanol, and 80 for water. The dielectric constant of media can vary widely so that electrostatic interactions are much weaker in water than in a non-polar medium. Not only the medium but also temperature affects the dielectric constant, for example, the dielectric constant of water is 80 at room temperature but is about 55 at 100 °C. Consequently, electrostatic interactions at high temperatures are stronger than at low temperatures. Although core cavities may hamper the stability of thermophilic proteins at high temperatures, surface salt bridges can compensate for the decreased stability.

Based on the statistical analysis of cavity properties in this study, vacant space with high flexibility of mesophilic proteins would be deleterious to the stability, and protein engineering to optimize the flexible surface cavity can be useful to increase the protein thermostability.

### 3.3. Examples of Engineering the Flexible Cavities in the Surface Areas to Improve Protein Thermostability

According to the statistical analysis of protein cavities between thermophilic and mesophilic proteins, the flexible cavities of proteins in boundary and surface can be engineered to improve protein thermostability. A few examples of protein engineering studies were reported for the engineering of flexible cavities in protein surfaces. The protein thermostability of mesophilic xylanase from *Bacillus circulans* was improved by engineering the flexible cavity-lining residues in the surface areas [26]. Residues with flexible motions in surface cavities were redesigned using a computational design approach to stabilize the local interactions of the surface cavities. Computational design of mesophilic xylanase was performed to search for more stable sequences that could strengthen the local interactions of the cavity-lining and the neighboring residues using the RosettaDesign algorithm [27]. Two surface cavities (cavity 6 and cavity 11) were selected for computational design and computationally designed eight mutants (F48Y, R49A, T50V, T147L in cavity 6 and D101N, G103F, R132A, R136A in cavity 11) were experimentally validated. Three thermostable single mutants (F48Y, T50V, and T147L) were obtained by mutating cavity-lining residues, and a more thermostable triple mutant (F48Y/T50V/T147L) engineered by a combination of the single mutants exhibited a 15-fold increase in the half-life of thermal inactivation [26]. In addition, more unstable regions in the wild-type xylanase were investigated by molecular dynamics simulations and target residues including the N52 residue were selected by analyzing flexibility changes. Computationally designed N52Y mutant showed a greater thermostabilization effect compared with three thermostable single mutants (F48Y, T50V, and T147L). Further combination of the computationally designed N52Y mutant with the triple mutations could lead to a more thermostable quadruple mutant (F48Y/T50V/N52Y/T147L) with a 60-fold increase in half-life than the wild-type [18]. MD simulations of the wild-type and quadruple mutant at 300 K and 330 K showed that the quadruple mutant was rigid at high temperature and the averaged RMSD difference of the quadruple mutant was smaller than that of the wild-type. This rational design of surface cavities indicates that sequential optimization of cavity-lining residues can dramatically increase the thermostability.

Cavity-filling mutation of the intramembrane protease GlpG from *E. coli* was performed to analyze the role of structural cavities in balancing stability and activity [28]. In total, eleven cavity-filling mutants in the five cavities from core to surface were designed to investigate the balance between stability and flexibility for optimal activity. MD simulation results performed in the previous study revealed that eight out of eleven single small-to-large mutations improved packing in the targeted cavities. Interestingly, experimental validation showed that two mutants i.e., M208I (cavity III) and A164L (cavity IV), could effectively reduce the volume of the two cavities by 30 to 60% and enhance the thermodynamic stability. In particular, the A164 mutant located in the surface area induced the largest stabilization (+0.9 ± 0.2 kcal/mol) among tested mutations. MD simulations and experiments of GlpG revealed that careful investigations on the protein packing and dynamics could improve the enzyme stability. Rational engineering of proteins based on structural analysis has been widely attempted to improve protein stability [29,30,31,32] and discovery and engineering of stable or active enzymes are very crucial for biological production of value-added chemicals [33,34,35,36,37]. Thus, the results obtained in this study can be applied to protein stabilization in various environments.

## 4. Conclusions

The role of cavity flexibility in protein thermostability was investigated by statistical analysis. Thermophilic proteins had fewer cavities in boundary and surface areas and in particular, cavities of mesophilic proteins in all areas were more flexible than those of thermophilic proteins. Compared to the thermophilic proteins, the flexible cavities of mesophilic proteins in boundary and surface can be deleterious to the stability. Recent studies on the cavity engineering of mesophilic proteins also corroborate that site-directed mutagenesis of cavity-lining residues in the surface area can contribute to the enhancement of thermostability. Based on these results, the rational or computational design of flexibility cavities in surface or boundary areas could be a good strategy to improve the thermostability of mesophilic proteins. In particular, the flexible cavity-lining residues within the surface or boundary regions can be altered through mutation to larger residues for cavity-filling, or they can be computationally designed to optimize their local interactions. In addition, the observed presence of more rigid cavities in the core regions of thermophilic proteins compared to those in mesophilic proteins may be associated with molecular motions at elevated temperatures. Consequently, additional molecular dynamics simulations could be conducted to investigate the role of cavities, not only in structural stability but also in catalytic motions, thereby balancing the stability and activity of enzymes.

## Figures and Tables

**Table 1:** Dataset of thermophilic and mesophilic proteins.

SCOP Fold Name	Thermophilic Proteins		Mesophilic Proteins	
	PDB	Organism	PDB	Organism
Adenine nucleotide alpha hydrolase-like	1V8FA	*Thermus thermophilus HB8*	1IHOB	*Escherichia coli*
Amionoacid dehydrogenase-like, N-terminal domain	1EUZA	*Thermococcus profundus*	1HRDA	*Clostridium symbiosum*
ATC-like	1ML4A	*Pyrococcus abyssi*	1D09A	*Escherichia coli*
Chorismate synthase, AroC	1Q1LA	*Aquifex aeolicus*	1QXOC	*Streptococcus pneumoniae*
DHS-like NAD/FAD-binding domain	1ICIB	*Archaeoglobus fulgidus*	1S5PA	*Escherichia coli*
Ferredoxin-like	1MROD	*Methanothermobacter marburgensis*	1E6YA	*Methanosarcina barkeri*
GroES-like	1RJWA	*Geobacillus stearothermophilus*	1LLUA	*Pseudomonas aeruginosa*
HAD-like	1F5SA	*Methanococcus jannaschii*	1NNLB	*Homo sapiens*
LDH C-terminal domain like	1IZ9A	*Thermus thermophilus*	1B8PA	*Aquaspirillum arcticum*
Macrodomain-like	1VHUA	*Archaeoglobus fulgidus*	1SPVA	*Escherichia coli*
NagB/RpiA/CoA transferase	1LK7A	*Pyrococcus horikoshii*	1M0SA	*Haemophilus influenzae*
Nucleotide-diphospho-sugar transferase	1LVWC	*Methanothermobacter thermautotrophicus*	1IIMA	*Salmonella enterica*
Phosphoglycerate kinase	1PHP	*Geobacillus stearothermophilus*	1VJCA	*Sus scrofa*
PLP-depedent transferase	1KL1A	*Geobacillus stearothermophilus*	1DFOA	*Escherichia coli*
S-adenosyl-L-methionine-depedent methyltransferase	1JQ3A	*Thermotoga maritima*	1IY9A	*Bacillus subtilis*
Subtilisin-like	1THM	*Thermoactinomyces vulgaris*	1NDOA	*Bacillus subtilis*
Thiamin diphosphate-binding fold (THDP-binding)	1UMCB	*Thermus thermophilus*	1OLSB	*Homo sapiens*
Thiolase-like	1J3NA	*Thermus thermophilus*	1IY9A	*Streptococcus pneumoniae*
YebC-like	1LFPA	*Aquifex aeolicus*	1KONA	*Escherichia coli*
YrdC/RibB	1PVWA	*Methanocaldococcus jannaschii*	1K4PA	*Magnaporthe grisea*

**Table 2:** The critical levels of t values for comparison of thermophilic and mesophilic proteins.

Df	t_0.1_	t_0.05_	t_0.025_	t_0.01_	t_0.005_
Inf (>30)	1.282	1.645	1.960	2.326	2.576

**Table 3:** Comparison of cavity properties in thermophilic and mesophilic proteins.

Proteins	Number of Proteins	Number of Cavities (Total ^a^/Average per Protein ^b^/Average per Residue ^c^)	Average Volume of Cavity (Å^3^)	Average Surface Area of Cavity (Å^2^)
Thermophilic proteins	20	369/18.45/0.056	26.65	45.53
Mesophilic proteins	20	355/17.75/0.056	27.51	46.20

^a^ Total cavity number of each group. ^b^ Average cavity number per protein = Total cavity number/20. ^c^ Average cavity number per residue = Total cavity number/Total residue number of each group. (Total residue number of thermophilic proteins = 6628; total residue number of mesophilic protein = 6346).

**Table 4:** Distribution of average cavity locations and flexibility between thermophilic and mesophilic proteins.

Structure Index (OSP Value)	Frequency						
	Thermo	SD ^a^	Flexibility ^b^	Meso	SD ^a^	Flexibility ^b^	*t*-test ^c^
Surface (0.00~0.250)	0.0081	±0.0202	−0.0034	0.0091	±0.0280	0.1985	−0.1233
Boundary (0.250~0.500)	0.7253	±0.1546	−0.2428	0.8025	±0.1203	−0.2047	−1.7621
Core (0.500~0.750)	0.2673	±0.1573	−0.6484	0.1884	±0.1099	−0.5111	1.8386

^a^ Standard deviation of the frequency. ^b^ B factor values of cavity-lining residues were first normalized and the average values of normalized B factor values in each structure index were used as a flexibility index. The higher the flexibility values, the higher the flexibility of cavities. ^c^ If the *t*-test value is more than 1.645, the probability that average cavity traits of thermophilic proteins is greater than those of mesophilic proteins if the given structure index is more than 95%. If the *t*-test value is lower than −1.645, the probability that the average cavity traits of thermophilic proteins is fewer than those of mesophilic proteins in a given structure is more than 95%.

## Data Availability

Data are contained within the article.

## References

[1] Zamani R., Rahpeyma S.S., Aliakbari M., Naderi M., Yazdanei M., Aminzadeh S., Khezri J., Haghbeen K., Karkhane A.A. (2023). Enhancing the Thermostability of Cellulase from *Clostridium thermocellum* via Salt Bridge Interactions. Biotechnol. Bioprocess Eng..

[2] Kim H., Lee U.-J., Lim G.-M., Kim J.-Y., Lee J., Song H., Kim E.-J., Kim J., Hwang N.S., Kim B.-G. (2023). Stability Enhancement of Target Enzymes via Tyrosinase-Mediated Site-Specific Polysaccharide Coating. Biotechnol. Bioprocess Eng..

[3] Chen L., Jiang K., Zhou Y., Zhu L., Chen X. (2022). Improving the Thermostability of α-Glucosidase from *Xanthomonas campestris* through Proline Substitutions Guided by Semi-rational Design. Biotechnol. Bioprocess Eng..

[4] Kumar S., Tsai C.J., Nussinov R. (2000). Factors enhancing protein thermostability. Protein Eng..

[5] Pack S.P., Yoo Y.J. (2005). Packing-based difference of structural features between thermophilic and mesophilic proteins. Int. J. Biol. Macromol..

[6] Arnold F.H., Wintrode P.L., Miyazaki K., Gershenson A. (2001). How enzymes adapt: Lessons from directed evolution. Trends Biochem. Sci..

[7] Eijsink V.G.H., Bjørk A., Gåseidnes S., Sirevåg R., Synstad B., Burg B.V.D., Vriend G. (2004). Rational engineering of enzyme stability. J. Biotechnol..

[8] Abraham T., Pack S.P., Je Yoo Y. (2005). Stabilization of *Bacillus subtilis* Lipase A by increasing the residual packing. Biocatal. Biotransformation.

[9] Dubey V.K., Jagannadham M.V. (2008). Roles for cavities in protein structure: New insights. Curr. Proteom..

[10] Frieden C. (1991). Effects of point mutations in a hinge region on the stability, folding, and enzymatic activity of *Escherichia coli* dihydrofolate reductase. Biochemistry.

[11] Glyakina A.V., Garbuzynskiy S.O., Lobanov M.Y., Galzitskaya O.V. (2007). Different packing of external residues can explain differences in the thermostability of proteins from thermophilic and mesophilic organisms. Bioinformatics.

[12] Gåseidnes S., Synstad B., Jia X., Kjellesvik H., Vriend G., Eijsink V.G.H. (2003). Stabilization of a chitinase from *Serratia marcescens* by Gly→Ala and Xxx→Pro mutations. Protein Eng..

[13] Sonavane S., Chakrabarti P. (2008). Cavities and atomic packing in protein structures and interfaces. PLoS Comput. Biol..

[14] Karshikoff A., Ladenstein R. (1998). Proteins from thermophilic and mesophilic organisms essentially do not differ in packing. Protein Eng..

[15] Szilágyi A., Závodszky P. (2000). Structural differences between mesophilic, moderately thermophilic and extremely thermophilic protein subunits: Results of a comprehensive survey. Structure.

[16] Pattabiraman N., Ward K.B., Fleming P.J. (1995). Occluded molecular surface: Analysis of protein packing. J. Mol. Recognit..

[17] Yokota K., Satou K., Ohki S.y. (2006). Comparative analysis of protein thermostability: Differences in amino acid content and substitution at the surfaces and in the core regions of thermophilic and mesophilic proteins. Sci. Technol. Adv. Mater..

[18] Joo J.C., Pack S.P., Kim Y.H., Yoo Y.J. (2011). Thermostabilization of Bacillus circulans xylanase: Computational optimization of unstable residues based on thermal fluctuation analysis. J. Biotechnol..

[19] Fleming P.J., Richards F.M. (2000). Protein packing: Dependence on protein size, secondary structure and amino acid composition. J. Mol. Biol..

[20] Tsodikov O.V., Thomas Record Jr M., Sergeev Y.V. (2002). Novel computer program for fast exact calculation of accessible and molecular surface areas and average surface curvature. J. Comput. Chem..

[21] Zhang H., Zhang T., Chen K., Shen S., Ruan J., Kurgan L. (2009). On the relation between residue flexibility and local solvent accessibility in proteins. Proteins: Struct. Funct. Bioformatics.

[22] Mendenhall W., Beaver R.J. (1991). Introduction to Probability and Statistics.

[23] Agarwal R., Shrestha U.R., Chu X.-Q., Petridis L., Smith J.C. (2020). Mesophilic Pyrophosphatase Function at High Temperature: A Molecular Dynamics Simulation Study. Biophys. J..

[24] Kalimeri M., Rahaman O., Melchionna S., Sterpone F. (2013). How Conformational Flexibility Stabilizes the Hyperthermophilic Elongation Factor G-Domain. J. Phys. Chem. B.

[25] Bogin O., Levin I., Hacham Y., Tel-Or S., Peretz M., Frolow F., Burstein Y. (2002). Structural basis for the enhanced thermal stability of alcohol dehydrogenase mutants from the mesophilic bacterium *Clostridium beijerinckii*: Contribution of salt bridging. Protein Sci..

[26] Joo J.C., Pohkrel S., Pack S.P., Yoo Y.J. (2010). Thermostabilization of Bacillus circulans xylanase via computational design of a flexible surface cavity. J. Biotechnol..

[27] Kuhlman B., Baker D. (2000). Native protein sequences are close to optimal for their structures. Proc. Natl. Acad. Sci. USA.

[28] Guo R., Cang Z., Yao J., Kim M., Deans E., Wei G., Kang S.-g., Hong H. (2020). Structural cavities are critical to balancing stability and activity of a membrane-integral enzyme. Proc. Natl. Acad. Sci. USA.

[29] Min K., Kim H., Park H.J., Lee S., Jung Y.J., Yoon J.H., Lee J.-S., Park K., Yoo Y.J., Joo J.C. (2021). Improving the catalytic performance of xylanase from Bacillus circulans through structure-based rational design. Bioresour. Technol..

[30] Min K., Kim H.T., Park S.J., Lee S., Jung Y.J., Lee J.-S., Yoo Y.J., Joo J.C. (2021). Improving the organic solvent resistance of lipase a from Bacillus subtilis in water–ethanol solvent through rational surface engineering. Bioresour. Technol..

[31] Jun C., Joo J.C., Lee J.H., Kim Y.H. (2014). Thermostabilization of glutamate decarboxylase B from Escherichia coli by structure-guided design of its pH-responsive N-terminal interdomain. J. Biotechnol..

[32] Goldenzweig A., Fleishman S.J. (2018). Principles of Protein Stability and Their Application in Computational Design. Annu. Rev. Biochem..

[33] Tran K.-N.T., Kumaravel A., Hong S.H. (2023). Impact of the Synthetic Scaffold Strategy on the Metabolic Pathway Engineering. Biotechnol. Bioprocess Eng..

[34] Matthay P., Schalck T., Verstraeten N., Michiels J. (2023). Strategies to Enhance the Biosynthesis of Monounsaturated Fatty Acids in Escherichia coli. Biotechnol. Bioprocess Eng..

[35] Woo J.-M., Kang Y.-S., Lee S.-M., Park S., Park J.-B. (2022). Substrate-binding Site Engineering of Candida antarctica Lipase B to Improve Selectivity for Synthesis of 1-monoacyl-sn-glycerols. Biotechnol. Bioprocess Eng..

[36] Khobragade T.P., Pagar A.D., Giri P., Sarak S., Jeon H., Joo S., Goh Y., Park B.-S., Yun H. (2023). Biocatalytic Cascade for Synthesis of Sitagliptin Intermediate Employing Coupled Transaminase. Biotechnol. Bioprocess Eng..

[37] Kang H., Sriramulu D.K., Lee S.-G. (2022). In silico Study on Binding Specificities of Cellular Retinol Binding Protein and Its Q108R Mutant. Biotechnol. Bioprocess Eng..

